# Occurrence of *Yersinia rohdei* among feral reindeer (*Rangifer t. tarandus*) and kelp gulls (*Larus dominicanus*) on the Sub-Antarctic island South Georgia

**DOI:** 10.1080/20008686.2018.1517582

**Published:** 2018-09-11

**Authors:** Jonas Bonnedahl, Charlotte Berg, Dan I. Andersson, Robert Söderlund, Ivar Vågsholm, Björn Olsen

**Affiliations:** a Department of Infectious Diseases, Kalmar County Council, Linköping University, Kalmar, Sweden; b Department of Animal Environment and Health, Swedish University of Agricultural Sciences, Skara, Sweden; c Department of Medical Biochemistry and Microbiology, Uppsala University, Uppsala, Sweden; d Department of Microbiology, National Veterinary Institute SVA, Uppsala, Sweden; e Department of Biomedical Sciences and Veterinary Public Health, Swedish University of Agricultural Sciences, Uppsala, Sweden; f Zoonosis Science Center, Department of Medical Sciences, Uppsala University, Uppsala, Sweden.

**Keywords:** Intestinal, kelp gull, rangifer, reindeer, sub-antarctic islands, yersinia

## Abstract

**Introduction:** During a research expedition in 2012, faecal samples were collected from feral reindeer and kelp gulls on the main island of South Georgia in the Sub-Antarctic region of the Atlantic. The samples were analysed for bacteria of the genus Yersinia with the aim of identifying isolates to the species level. **Materials and Methods:** A total of 11 reindeer samples and 26 Kelp Gull samples were retrieved from the location of Stromness, kept refrigerated and cultivated for gram-negative bacteria. **Results:** Three of the samples showed growth of Yersinia rohdei, as confirmed by biochemical typing, MALDI-TOF and 16S rRNA sequencing. The isolates were indistinguishable from each other by 16S sequencing, and differed by a single base pair from the type strain of Y. rohdei. **Discussion:** The Yersinia genus contains well-known pathogens of significance to both human and veterinary medicine, but the public health and animal health relevance of Y. rohdei is unknown. Although it is clear that Y. rhodei is present in the south Georgian biotope, its importance and relevance for biological diversity is unknown, as is if this presence is merely a reflection of human activities.

## Introduction

The South Georgia Island of the Sub-Antarctic southern Atlantic region is remote and isolated, and naturally harbour no terrestrial mammals. Nevertheless, the island is one of the most important marine wildlife breeding areas in the world with over 30 million breeding pairs of birds including endemic taxa such as South Georgia Pipit (*Anthus antarcticus*) and South Georgia Pintail (*Anas georgica georgica*). The islands also serve as breeding grounds for marine mammals, such as Antarctic Fur Seals (*Arctocephalus gazella*) and Southern Elephant Seals (*Mirounga leonina*).

Furthermore, a number of introduced species are or have been present on the islands, such as reindeer (*Rangifer t. tarandus*), brown rats (*Rattus norvegicus*) and house mice (*Mus musculus*). While the rats and mice have been accidentally introduced, reindeer were deliberately brought to the island. The first introduction was made by Norwegian whalers in 1911, followed by two more introductions in 1912 and 1925 [1], to serve as food for the whaling communities. After the end of the whaling era in the 1960’s the reindeer herds were left to range and proliferate freely on South Georgia [2]. No natural enemies of reindeer are found in the region [2].

As reindeer are not predators and hence do not prey on eggs and chicks, they may not be perceived as an obvious threat to the native wild bird populations, like the rats and mice are. However, they indirectly posed a threat to several species on South Georgia, because of their large number and grazing habits [3]. As there are no native terrestrial grazing animals at all on South Georgia [2], the vegetation on the island has not evolved under grazing conditions and cannot withstand heavy grazing pressure. The presence of reindeer has therefore led to extensive soil erosion and negatively affected the biodiversity of plants and birds as a result of overgrazing [2,4], and an official eradication programme was established in the early 2010’s. As a result, a total of almost 7000 reindeer were shot or slaughtered for meat production during 2013–2015 [5].

The kelp gull (*Larus dominicanus*) is a large gull found in the Southern hemisphere, along the coastal areas of South America, southern Africa, Australia and the Sub-Antarctic islands [6]. They mainly feed on small prey but are also frequent scavengers, which means that they will come in contact with, and ingest, a large variety of microbes present in the environment.

Research on vertebrate-associated bacteria in these habitats is rare, but a couple of studies have focused on human-associated and zoonotic agents in wildlife and the environment around research stations [7,8] and also in more remote locations [9] .*Yersinia* spp have previously been isolated in samples from semi-domesticated reindeer in Norway and Finland [10], and we therefore hypothesized that such microbes may be present also in the population on South Georgia.

The bacterial genus *Yersinia*, including at least 11 species, belongs to the family *Enterobacteriacae*, being Gram-negative, facultative anaerobes. The most well-known human pathogen species of this genus is *Y. pestis* (plague). Furthermore, *Y. enterocolitica* and *Y. pseudotuberculosis* are enteropathogens with relevance to both human and veterinary medicine, and can be found in both domestic and wild birds and mammals, including reindeer [11]. Such infections in humans are mainly considered to be foodborne [12,13]. *Yersinia rohdei has* previously been isolated from several mammal species, including humans with and without symptoms of gastrointestinal disease, healthy dogs [14] and reindeer [10,15], and from migratory birds [16]. In summary, *Y. pestis, Y. enterocolitica* and *Y. pseudotuberculosis* are associated with human and animal diseases, and considered zoonotic [17]. The other *Yersinia* spp. including *Y. rohdei*, are considered to be environmental species but may have opportunistic pathogen properties [18], or cause disease in fish. Hence, the remaining eight (or more) *Yersinia* spp., absent of classical *Yersinia* virulence markers, may in some cases still cause disease although the mechanisms have not been fully understood [19].

The overall aim of this research expedition was to isolate and characterize zoonotic agents and screen for antibiotic resistant bacteria in marine birds [9], but samples were taken also from mammals, when possible. Our aim in this paper was to identify *Yersina* isolates to species level and to provide a description of the *Yersinia* strains found in such an isolated population as the South Georgian reindeer and in its habitat.

## Material and methods

### Sampling and cultures

Samples were collected in the Sub-Antarctic region of the Atlantic in 2012. Fresh faecal samples for bacterial cultures were obtained from the ground or as cloacal swabs from apparently clinically healthy birds and mammals. Intervention with any species present in the areas of sampling was kept to a minimum to safeguard wildlife welfare. High level of biosecurity was applied, to minimize the risk of introducing new microbes into these relatively pristine areas, and appropriate protective gear was used by the staff retrieving the samples. The samples were obtained with sterile cotton swabs, which were stored in Amies charcoaled medium (Copan Italia S.P.A.), and refrigerated at 5–10°C for a duration of 1–3 weeks until arrival at the laboratory in Sweden where they were cultured within 24 h [9].

For the entire study, a total of 360 samples were retrieved from southern Argentina, the Falkland Islands, South Georgia and the Antarctic Peninsula and its surrounding archipelago. From the location of Stromness (54° 9′ 36″ S, 36° 42′ 41.76″ W) on South Georgia a total of 11 reindeer samples, 26 Kelp Gull samples, 20 Giant Storm Petrel (*Macronectes giganteus*) samples, 9 Brow Skua (*Stercorarius antarcticus*) samples and 1 fur seal sample were retrieved. All samples from all locations were analysed for presence of, among other microbes, bacteria of the genus *Yersinia*. For a list of locations and a complete list of avian species sampled within the entire study, please see Jansson and and co-workers [9].

### Isolation of Yersinia strains

Faecal material from all 360 samples were inoculated with 2 ml brain heat infusion broth (BHI-broth; Becton, Dickinson, USA), supplemented with vancomycin (16 mg/L, ICN Biomedicals Inc., USA) and incubated for 18–24 hours at 36°C for enrichment. From the incubated BHI-broth, 1 µl of material were streaked on an Uriselect agar plate (Bio-Rad Laboratories, France) to screen for *Enterobacteriaceae* with a special focus on *Escherichia coli*. All pink, purple or red colonies growing on the Uriselect agar plate was then furthered confirmed to species level by MALDI-TOF.

### Sequence analysis of 16S rRNA genes

Partial 16S rRNA gene sequencing was performed as previously described [20], producing seven sequence reads from each 1447 bp amplicon (excluding the initial primers). Sequences were assembled and manually verified using BioNumerics 7.6. Corresponding 16S sequences from other isolates of *Y. rohdei* and several type strains of *Yersinia* species were downloaded from GenBank, and compared to the isolates from this study. A phylogenetic tree was created in BioNumerics 7.6 using the neighbour-joining algorithm with Kimura 2-parameter correction of distances.

## Results

Of all the 360 samples covered by the main study, only three were positive for *Yersinia* spp. Since the initial isolation was focused on *Escherichia coli* and not optimized for *Yersinia spp*. we cannot completely rule out that more *Yersina* may have been present. These three samples were all taken at the location of Stromness on South Georgia, one from a reindeer and two from Kelp Gulls. None of the other samples from the same location contained any *Yersinia* under this isolation condition.

### Sequence analysis of the 16S rRNA genes

**Figure 1.**
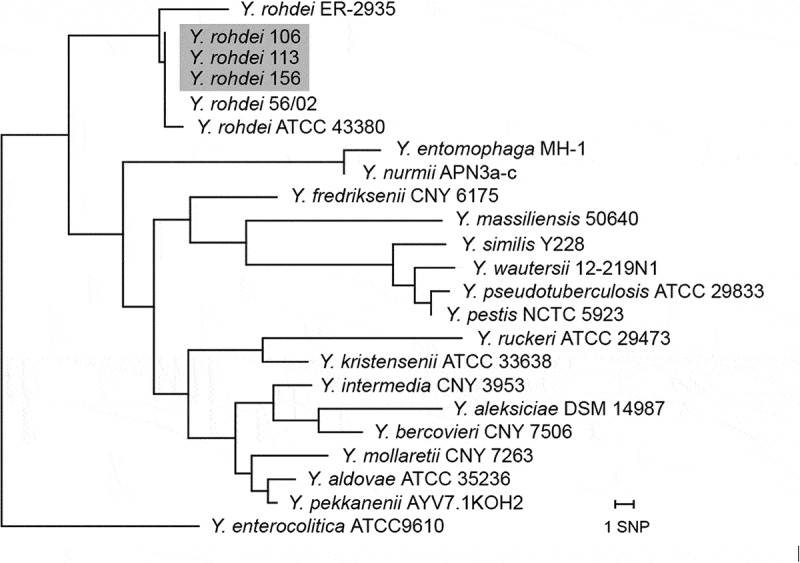
Phylogenetic tree based on partial 16S rRNA sequencing of *Y. rohdei* isolates from reindeer and kelp gull investigated in the present study (highlighted) compared to other *Y. rohdei* isolates and the type strains of other *Yersinia* species.

All three isolates had identical partial 16S rRNA sequences. Four ambiguous positions were found, two A/G and two C/T, likely due to the presence of multiple rRNA operons with different sequences in the *Y. rohdei* genome. Comparison with publicly available 16S rRNA sequences from *Y. rohdei* revealed an exact match with an isolate from sheep in the UK 2002 (56/02). The isolates were also highly similar to the *Y. rohdei* type strain ATCC 43,380, differing by a single base pair. Comparison with publicly available 16S rRNA sequences from the type strains of several *Yersinia* species showed low similarity with the isolates from this study (Figure 1). The assembled sequences were deposited in the European Nucleotide Archive (ENA) with study accession PRJEB21628, and sequence accessions LT899396-LT899398.

## Discussion

In this paper, we describe a strain of *Yersinia rohdei*. To the best of our knowledge these *Yersinia* isolates are the first to be described from the region of South Georgia.

*Yersinia* has previously been isolated from reindeer [10,20–23], and from Kelp Gulls [24]. In one of the reindeer studies [10] *Yersinia* species were isolated from 108 samples, consisting mainly of *Y. kristensenii* (n = 72) and *Y. enterocolitica* (n = 29), and only two cases of *Y. rohdei*. The authors concluded that the *Yersinia* strains identified in that study were of very low virulence and pathogenicity, which applied also to other bacteria isolated in that study, and hence posed very little risk to human and animal health. In a similar study carried out in Northern Norway no *Yersinia* spp. were isolated from any of the faecal samples [25]. In a study of 57 migratory bird species sampled in Sweden in the year 2000, a total of 60 out of 468 samples were positive for *Yersinia* spp., but only 3 of these were *Y. rohdei*, while a majority were *Y. enterocolitica, Y. intermedia* or *Y. fredriksenii* [16].

### Arctic/Antarctic region

The Kelp gulls show opportunistic, scavenging feeding habits and are hence likely to be exposed to a variety of bacteria, perhaps including *Yersinia* spp. We cannot exclude bacterial transmission in the other direction, i.e. from gulls to reindeer, through pasture contamination, but it appears to be a less likely option. Kelp gulls along the Patagonian coast, in closer proximity to humans and domestic animals, have previously been shown to harbour *Yersinia enterocolitica* bacteria at low prevalences (1 positive sample out of 60), and also other pathogen microbes [24]. Similarly, Kelp gulls in Ushuaia in southern Argentine, have been demonstrated to harbour antibiotic resistant Enterobacteriaceae including a variety of Extended-spectrum β-lactamase-producing strains [26].

The South Georgian reindeer population was completely eradicated during the eradication operation in 2013–2015 and hence no further sampling can be made from these animals. It is unclear if the scientific sampling during the second phase of the eradication programme in 2014, covering the investigation into the diversity of the gut flora [5] included analyses of presence of *Yersinia*. However, the reindeer genetic resource still prevails since a small number of calves were translocated to the Falkland Islands in 2001–2003 for production purposes [1]. This means that not only is this particular group of reindeer, isolated since the 1920’s, still existing although at a new location, but also that some of the microbes harboured by these ruminants may still be isolated. It is not known how the intestinal flora of the animals on South Georgia after almost 100 years in the Southern hemisphere differed from that of the original reindeer population in Scandinavia. Neither is it known how it may have been changed after the transfer to the Falkland Islands, which have a different flora and fauna, including the presence of domestic animals, compared to South Georgia. Furthermore, it is to our knowledge not known if the reindeer currently kept on the Falkland Islands harbour *Yersinia* bacteria.
